# Key role of the 3' untranslated region in the cell cycle regulated expression of the *Leishmania infantum *histone H2A genes: minor synergistic effect of the 5' untranslated region

**DOI:** 10.1186/1471-2199-10-48

**Published:** 2009-05-21

**Authors:** Daniel R Abanades, Laura Ramírez, Salvador Iborra, Ketty Soteriadou, Victor M González, Pedro Bonay, Carlos Alonso, Manuel Soto

**Affiliations:** 1 Centro de Biología Molecular Severo Ochoa, Departamento de Biología Molecular, Universidad Autónoma de Madrid, CSIC-UAM, Nicolás Cabrera 1, 28049 Madrid, Spain; 2 Unidad de Inmunología Viral, Centro Nacional de Microbiología, Instituto de Salud Carlos III, Crta. Pozuelo Km 2, 28220 Majadahonda, Madrid, Spain; 3 Laboratory of Molecular Parasitology, Hellenic Pasteur Institute, 127 Vas. Sophias, 115 21 Athens, Greece; 4 Departamento de Bioquímica-Investigación, Hospital Ramón y Cajal, 28034 Madrid, Spain

## Abstract

**Background:**

Histone synthesis in *Leishmania *is tightly coupled to DNA replication by a post-transcriptional mechanism operating at the level of translation.

**Results:**

In this work we have analyzed the implication of the 5' and 3' untranslated regions (UTR) in the cell cycle regulated expression of the histone *H2A *in *Leishmania infantum*. For that purpose, *L. infantum *promastigotes were stably transfected with different plasmid constructs in which the *CAT *coding region used as a reporter was flanked by the 5' and 3' UTR regions of the different *H2A *genes. We report that in spite of their sequence differences, histone *H2A *5' and 3' UTRs conferred a cell cycle dependent pattern of expression on the CAT reporter since *de novo *synthesis of CAT increased when parasites enter the S phase. Using one established *L. infantum *cell line we showed that CAT expression is controlled by the same regulatory events that control the endogenous histone gene expression. Thus, although we did not detect changes in the level of *CAT *mRNAs during cell cycle progression, a drastic change in the polysome profiles of *CAT *mRNAs was observed during the progression from G1 to S phase. In the S phase *CAT *mRNAs were on polyribosomal fractions, but in the G1 phase the association of *CAT *transcripts with ribosomes was impaired. Furthermore, it was determined that the addition of just the *H2A *3' UTR to the *CAT *reporter gene is sufficient to achieve a similar pattern of post-transcriptional regulation indicating that this region contains the major regulatory sequences involved in the cell cycle dependent expression of the *H2A *genes. On the other hand, although CAT transcripts bearing the *H2A *5' alone were translated both in the G1 and S phase, higher percentages of transcripts were detected on polyribosomes in the S phase correlating with an increase in the *de novo *synthesis of CAT. Thus, it can be concluded that this region also contributes, although to a minor extent than the 3' UTR, in the enhancement of translation in the S phase relative to the G1 phase.

**Conclusion:**

Our findings indicate that both, the 5' and the 3' UTRs contain sequence elements that contribute to the cell cycle expression of *L. infantum *H2A. The 3' UTR region is essential for cell cycle dependent translation of the *L. infantum *H2A transcripts whereas the 5' UTR has a minor contribution in their S phase dependent translation.

## Background

Genes encoding histones are one of the best studied examples of cell cycle regulated genes in eukaryotic organisms. Multiple levels of control operate in the cell in order to restrict the synthesis of histones to the S phase of the cell cycle. In mammalian cells, cell-cycle dependent histone genes are regulated at the level of transcription, pre-mRNA processing and mRNA stability [1]. Processing and stability are controlled by the interaction between a stem loop binding protein (SLBP) and a conserved stem-loop element present in the 3' UTRs of the histone transcripts, that are not polyadenylated [2,3]. Also, SLBP stimulates translation of histone mRNAs during the S phase of the cell cycle [4]. In yeast and other lower eukaryotes histone mRNAs are polyadenylated and do not terminate with these stem-loop structures. In these organisms the S phase dependent expression of histone genes is controlled mainly at the transcription level, but also post-transcriptional regulation results in the preferential degradation of histone mRNAs outside of the S phase in *Saccharomyces cerevisiae *[5-7].

As it occurs in lower eukaryotes the trypanosomatid histone mRNAs are polyadenylated (reviewed by ([8]) but histone gene transcription appears to be constitutive as it is the case for most genes, in correlation to the lack of regulated RNA polymerase II promoters described for these organisms [9]. Although histone gene transcription is not coordinated with DNA replication, in *Trypanosoma cruzi *[10] and *Leishmania infantum *[11] the core histone biosynthesis is tightly coupled with DNA synthesis during the cell cycle. The molecular mechanism controlling the cell-cycle regulated expression of histone genes differs among trypanosomatids. In *Trypanosoma sp*. mRNA stability plays a major role in histone expression (reviewed by [12]). In *T. cruzi *the use of inhibitors of DNA synthesis has shown that the histone mRNA levels are coupled to the rate of DNA synthesis [13,14] and that the histone mRNA levels peak during the S phase of the cell cycle [10,15]. Similarly, in *Trypanosoma brucei *the abundance of histone *H2B *mRNAs decreased after growing parasites in the presence of the DNA synthesis inhibitor hydroxyurea (HU) [16] making histone mRNAs undetectable out of the S phase by *in situ *hybridization analysis [17].

Contrary to the situation in *Trypanosoma sp*., the histone mRNA levels in *Leishmania *do not decrease after treatments with DNA synthesis inhibitors [18-20]. Histone biosynthesis is regulated by a mechanism involving a translational control that is exerted on histone mRNAs in the absence of DNA synthesis [21]. Even though there are no significant variations in the steady-state levels of *Leishmania *histone mRNAs along the cell cycle, drastic changes in the polysome profiles of histone mRNAs were observed during the progression from the G1 to the S phase. Thus, in the G1 phase the association of the histone transcripts with ribosomes was impaired and in the S phase histone mRNAs are present in polyribosome fractions [11]. A regulatory model in which the cell cycle synthesis of histones in *Leishmania *is controlled through reversible interactions of protein factors that modulate translation of histone mRNAs has been proposed [11]. This mechanism would involve the existence of *cis*-regulatory signals in the histone mRNAs where specific RNA binding proteins are bound.

In all trypanosomatids protein coding genes are organized as large clusters on the same DNA strand and transcribed from undefined promoters as large polycistronic precursor RNAs. Polycistronic transcripts mature by *trans*-splicing which results in the addition of a short capped leader (39-mer) to the 5' end of mRNAs and by 3' processing that includes cleavage and polyadenylation [9]. *Trans*-splicing and polyadenylation are coupled mechanistically and the poly(A) site selection seems to be specified by the position of the downstream splice acceptor site [22]. Given that regulated gene expression in *Leishmania *has been related with post-transcriptional events mainly involving sequences present in the 5' and 3' UTRs [23], in the present work we have analyzed the involvement of these regions in the cell cycle dependent expression of the *L. infantum *H2A genes. For that purpose the expression of a reporter gene was analyzed in parasite cell lines stably transfected with different plasmid constructs containing combinations of the *Leishmania H2A *5' and 3' UTRs.

## Results and discussion

### The 5' and 3' UTR of the *L. infantum *H2A genes confer cell cycle regulated expression to the CAT reporter gene

**Figure 1:**
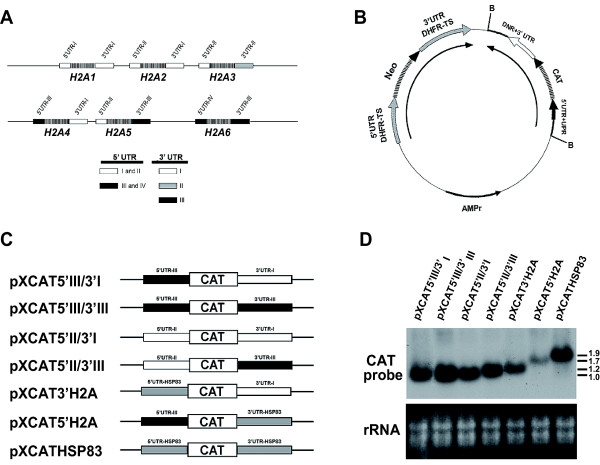
**DNA constructs**. (A) Schematic representation of the two *Leishmania infantum *H2A loci. The names of the genes are indicated below the maps. The locations of the different 5'(I-IV) and 3' UTR (I-III) are indicated on the diagram. Due to the sequence similarity 5'UTR-I and -II are represented as white boxes and 5'UTR-III and -IV are represented as black boxes. These sequences are in the EMBL databases under the accession numbers AJ419625 and AJ419627. (B) Generic representation of the pX63NEO recombinant vector. Position of the *Bam*HI cut sites employed in the cloning are indicated as B. Positions of the 5'UTR + upstream regions (UPR) and 3' UTR + downstream regions (DNR) relative to *CAT *gene coding region are indicated. Transcriptional directions of the *NEO *and *CAT *genes are as indicated. (C) Schematic outlines of the constructs used in this work indicating the name of the clone and the type of 5'UTR+UPR and 3'UTR+DNR included around the *CAT *coding region. (D) Northern blots of RNA from promastigotes stably transfected with the different constructs and hybridized with radiollabeled oligonucleotide complementary to the 3' terminal region of *CAT *gene coding region. Ethidium bromide staining of the corresponding gel is also shown (rRNA panel).

Two H2A loci are present in the *L. infantum *genome each one located in a different chromosome (Chr21 and Chr29 [24]) (Fig. 1A). The three gene copies located in each locus although highly conserved in the coding region present major sequence differences in the 5' UTRs and in the 3' UTRs. There are four types of 5' UTRs, named 5' UTR-I to -IV, with remarkable sequence conservation between the 5' UTR-I and 5' UTR-II and between the 5' UTR-III and 5' UTR-IV, respectively [25]. On the other hand, in the H2A loci there are three types of 3' UTR that are completely divergent in their nucleotide sequence (namely 3' UTR-I, -II and -III). An analysis of the relative steady-state levels of the different *L. infantum H2A *mRNAs revealed that transcripts containing the 3' UTR-I and 3' UTR-III account for the majority of the *H2A *transcripts in logarithmic-phase growing promastigotes. In addition, the 3' UTR-I containing transcripts are approximately 7-fold more abundant than those containing the 3' UTR-III. Very low steady-state levels were found for transcripts that contain the 3' UTR-II [25]. For that reason 3'UTR-II has been excluded in our analysis.

Constructs containing each one of the two expressed 3' UTR (-I and -III) combined with each one of the 5' UTR types (-II and -III) were cloned downstream and upstream to the *CAT *reporter gene, respectively. All the constructs were subcloned into the trypanosomatid expression vector pX63NEO in opposite direction with respect to the *NEO *gene that is controlled by the regulatory regions of the *DHFR-TS *gene (Fig. 1B). Thus, the reporter gene may be expressed independently of the regulatory regions included in the pX63NEO plasmid [26]. A schematic representation of the different constructs containing the *CAT *reporter is shown in Fig. 1C. Sequences located upstream of the 5' UTR and downstream of the 3' UTR were included for correct trans-splicing and polyadenylation of the *CAT *transcripts (see Methods section for a detailed description). Northern blot analysis of stably transfected cell lines containing plasmids pXCAT5'III/3'I (regulatory regions of gene *H2A4*), pXCAT5'II/3'III (regulatory regions of gene *H2A5*) and plasmids pXCAT5'III/3'III and pXCAT5'II/3'I (chimeric genes in which regulatory regions from gene *H2A4 *and *H2A5 *were combined) gave rise to a single *CAT *transcript of approximately 1 Kb (Fig 1D). This size is in accordance with a correct RNA processing as expected from the length of the different *H2A *mRNAs [25]. The boundaries of the 5' UTRs and 3' UTRs of the different *CAT *transcripts were defined by an RT-PCR approach (see Methods). The presence of the H2A 3' UTRs seems to confer higher stability to the reporter transcripts since the steady-state level of *CAT *RNAs in the pXCAT5'H2A cell line was lower when compared to the other ones. In this cell line a single band of approximately 1.7 Kb was observed, in accordance with the boundaries of the 5' UTR of gene *H2A4 *and 3' UTR of gene *HSP83 *included in the construct determined by RT-PCR (see Methods). A cell line stably transfected with plasmid pXCATHSP83 (composed of the 5' and 3' regions of *HSP83 *gene controlling *CAT *expression) used as control, gave rise to single band of approximately 1.9 Kb (Fig. 1D), as expected after the processing of *HSP83 *UTRs [27,28] (see Methods).

**Figure 2:**
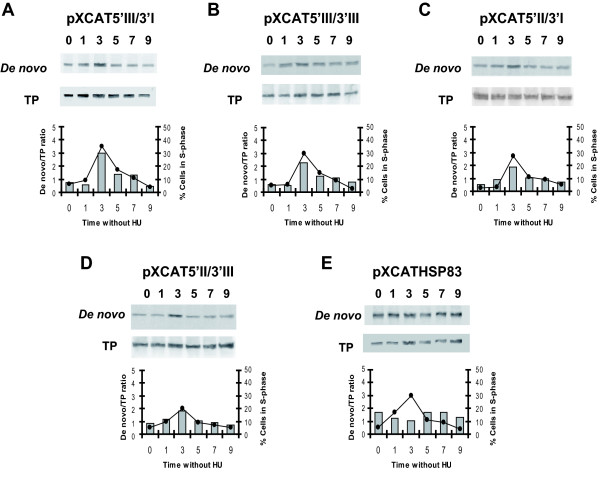
**Expression of CAT along the cell cycle in the different transfected *Leishmania *cell lines**. Protein extracts from ^35^S-labelled *L. infantum *promastigotes transfected with the indicated constructs and treated with 5 mM HU either for 12 h (lane 0) or at the indicated time period (in h) after removal of the drug, were used to immunoprecipitate CAT. After immunoprecipitation, protein samples were separated by SDS/PAGE and transferred on to a nitrocellulose membrane. After autoradiographic exposure (*De novo *panels), membranes were incubated with the anti-CAT antibody to reveal the total amount of CAT present in the samples (TP panels). Graphics show the ratio between *de novo *synthesis of CAT and total amount of CAT determined by densitometric quantification (columns) and the percentage of cells in the S phase of the cell cycle determined by flow cytometric analysis (line). Data correspond to one representative experiment of three independent assays with similar results.

**Figure 3:**
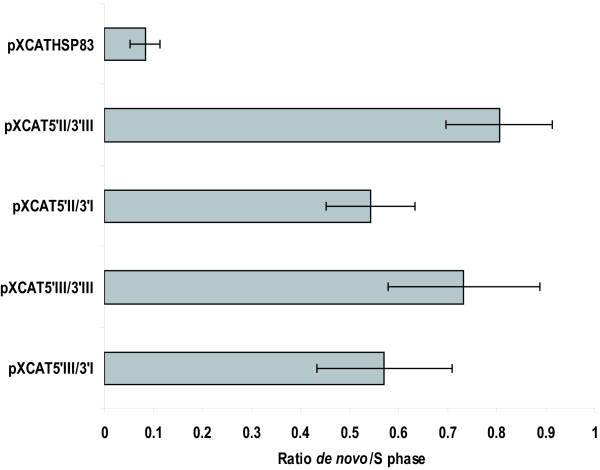
**Ratio between the *de novo *CAT synthesis and number of cells in the S phase of the cell cycle**. Ratio between the *de novo *CAT synthesis (normalised to the total amount of CAT) and the number of cells in the S phase (3 h after HU removal) determined by three independent assays. The assays were performed for the transfectants bearing both the 5' and 3' UTR H2A gene regulatory regions as well as for the transfectant with the HSP83 gene UTRs

Fig. 2 summarizes the studies on the effect of the flanking sequences of *H2A *genes on the cell cycle dependent expression of the *CAT *gene. For that purpose the different transfected promastigote cell lines were treated for 12 h with hydroxyurea (HU) and after drug removal cells were collected at different time periods and stained with propidium iodide to determine the percentage of cells in the different cell-cycle phases. HU removal resulted in a semi-synchronous entry into the cell cycle in *L. infantum *promastigotes, in line with previous reports [11]. At the same time the rate of the *de novo *synthesis of CAT was determined by immunoprecipitation with an anti-CAT polyclonal antibody on protein extracts labelled with [^35^S]methionine. The autoradiograph exposure of the blot showed that ^35^S-labelling of CAT reached a maximum at 3 h and then decreased to initial levels 9 h after HU removal for all the cell lines containing the regulatory regions of the different *H2A *genes (Fig. 2A–D). Densitometric analysis of the results showed a 3- to 4-fold increase at the 3 h point, correlating to the maximum percentage of cells in the S phase determined by flow cytometry. On the other hand, the amount of radioactivity bound to the immunoprecipitated CAT in the cell line transfected with pXCATHSP83 plasmid did not show the cell cycle specific expression profile observed for the other constructs (Fig. 2E). Since in the context of these experiments variations were observed in the percentage of cells in the S phase (3 h after HU removal) three independent experiments were performed with all the cell lines showed in Fig. 2, and the ratio between the *de novo *synthesis (normalised to the total amount of CAT) and cells in the S phase (3 h after HU removal) was determined. A significant increase was observed in this ratio when comparing the transfectants bearing the different H2A gene regulatory regions to the transfectant with the HSP83 gene UTRs (Fig. 3). In Fig. 3 it is also shown that transfectants bearing the 3' UTR-III, independently of the 5' UTR, have a moderately higher ratio which is indicative of a higher translational potential.

Thus, it can be concluded that the presence of the *H2A *regulatory regions confers a cell cycle dependent expression of CAT as it has previously been reported for *Leishmania *histone synthesis [11].

### Cell cycle dependent expression of CAT is regulated at the translational level

**Figure 4:**
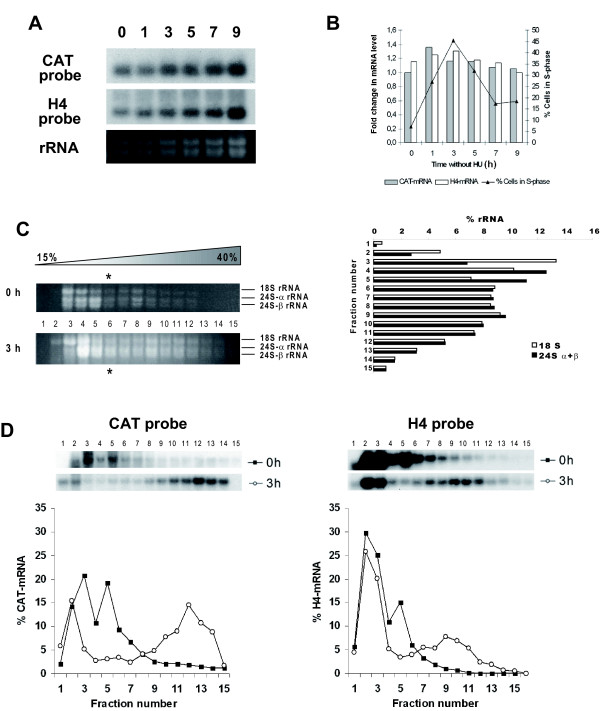
**Cell cycle dependent expression of CAT is regulated at the translational level**. (A) Northern-blot analysis of total RNA samples for promastigotes transfected with pXCAT5'III/3'I treated with 5 mM HU either for 12 h (lane 0) or at the indicated time (in h) after removal of the drug. The blot was sequentially probed with an oligonucleotide reverse and complementary to the 3' end of the *CAT *coding region and with a cDNA coding for *L. infantum *H4. Ethidium bromide staining of the corresponding gel is also shown (rRNA panel). (B) Graphic showing the ratio between the densitometric values of the blots hybridized with CAT probe (black columns) or H4 probe (white columns) and the rRNA bands revealed by ethidium bromide staining of the gel. Also the percentage of cells in the S phase of the cell cycle determined by flow cytometric analysis (black line) is shown. (C) Cytoplasmic extracts from *L. infantum *promastigotes transfected with pXCAT5'III/3'I and treated with 5 mM HU either for 12 h or 3 h after drug removal were separated on 15–40% sucrose linear gradients and RNA was purified and resolved in a 1% agarose-formaldehyde gel. The ethidium bromide staining of the gel is shown. Migration of 18 S, 24 S-α and 24 S-β are indicated. A graphic showing the densitometric values of the gel obtained 3 h after drug removal is included. The migration of the 80S particles has been marked in the gradients by an asterisk. (D) RNA from these gels were transferred on to nylon membranes and sequentially probed with the CAT and H4 probes. The autoradiographic exposure of the blot and the densitometric analysis is shown. Results are plotted as percentages of the total signal to allow direct comparison of the polysomal profiles. Data correspond to one representative experiment of three independent assays with similar results.

In order to analyze where the control of *CAT *gene expression is exerted, two different assays using promastigotes stably transfected with pXCAT5'III/3'I construct (containing the regulatory regions of gene *H2A4*) were performed. Firstly, a Northern blot of RNA isolated at various time points after HU release was hybridized with a CAT probe (Fig. 4A). Quantification of the obtained radioactive signals normalized to all ribosomal rRNA bands indicated that *CAT *mRNA levels remain constant throughout the cell cycle (Fig. 4B). Secondly, we analyzed the distribution of *CAT *mRNAs on polyribosomes during the progression from G1 and S phase. For that purpose the polysomal profile of the *CAT *transcripts in promastigotes at phase G1 (parasites treated for 12 h with 5 mM HU) and mid-S phase (3 h after HU release) was studied by sucrose gradient centrifugation of cytosolic extracts and Northern blotting. According to the rRNA species distribution on the gradients deduced from the ethidium bromide staining, fractions 1–5 should be considered to be free of functional ribosomes, since they either do not contain rRNAs or the rRNAs are not in equimolecular amounts. Equimolarity of the three larger 18 S, 24 S-α and 24 S-β rRNAs composing the *L. infantum *ribosome [29] was observed between aliquots 6–15 that correspond with the polyribosomal fractions (Fig. 4C). The distribution of the *CAT *mRNAs along the sucrose gradient fractions is shown in Fig 4D. During the G1 phase, *CAT *transcripts concentrate on the top of the gradient showing a peak out of the polysome fractions. On the contrary, when 50% of the cells were in the S phase *CAT *mRNA was detected in the bottom fractions. In particular, a secondary peak containing 57% of the *CAT *mRNAs was observed in the polyribosomal fractions. A similar pattern was obtained when blots were re-hybridized with a H4 probe although the percentage of *H4 *mRNAs in the polyribosome fractions was lower (41% of the *H4 *mRNAs) and the secondary peak was located in the central fractions of the sucrose gradient. The same results showed for H4 were obtained when a H2A coding region probe was tested in these blots (data not shown). Differences in the translational degree observed in the *CAT *mRNAs polysome profiles in the S phase relative to the endogenous H4 or H2A could be related to the influence of the CAT coding region on the secondary structure of the mRNAs since it has been recently described that the coding regions of different reporter genes may affect the translational efficiency in kinetoplastids [30]. Together, these results demonstrate that the cell-cycle related expression of CAT is controlled in the transfected parasites by the same regulatory events that control the expression of the histone genes.

### Histone H2A 3' UTR is involved in S phase translational regulation

To examine the role of the histone 3' UTR regions in the control of mRNA translation we made a new construct, pXCAT3'H2A, in which the *CAT *reporter coding region was located between the 5' regulatory regions of *HSP83 *gene and the 3' regulatory regions of *H2A4 *gene (Fig. 1C). Northern blot analysis of stably transfected cells lines containing this construct gave rise to a single *CAT *transcript of approximately 1.2 Kb as expected after correct 5' and 3' processing or the mRNA (Fig. 1D). The steady state level of these transcripts remained constant throughout the cell cycle (data not shown) and had similar levels to what was observed for the cell line transfected with the plasmid containing both *H2A4 *gene regulatory regions (pXCAT5'III/3'I) (Fig. 1D).

**Figure 5:**
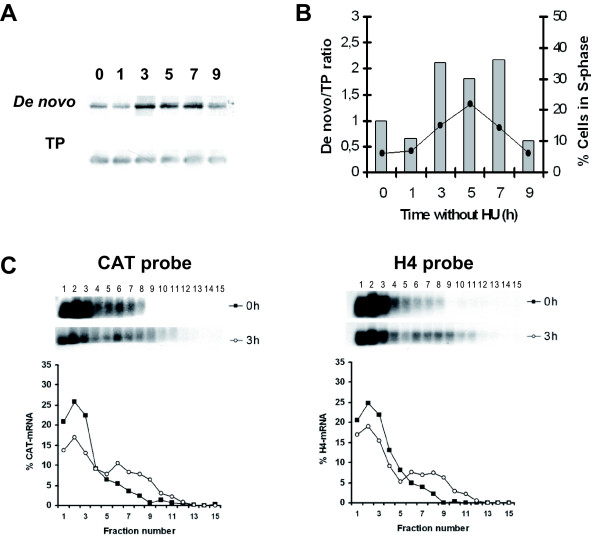
**Effect of the *H2A4 *3' UTR on regulation of CAT expression**. (A) Protein extracts from ^35^S-labelled *L. infantum *promastigotes transfected with pXCAT3'H2A and treated with 5 mM HU either for 12 h (lane 0) or at the indicated time period (in h) after removal of the drug, were used to immunoprecipitate CAT. After immunoprecipitation, protein samples were separated by SDS/PAGE and transferred on to a nitrocellulose membrane. Authoradiography (*De novo *panel) or Western blot with anti-CAT antibodies to reveal the total amount of CAT present in the samples (TP panel) is shown. (B) Densitometric analysis showing the ratio between de novo synthesis of CAT and total amount of CAT (columns). The percentage of cells in the S phase is also included (black line). The assay was repeated twice with similar results. (C) Northern blots prepared with RNA samples separated by sucrose gradients from promastigotes transfected with pXCAT3'H2A and treated 12 with HU or 3 h after drug removal were sequentially hybridized with the CAT and the H4 probe. The autoradiographic exposure of the blots and the densitometric analysis is shown. Data correspond to one representative experiment of three independent assays with similar results.

On the other hand, a good correlation was observed between the percentage of cells in the S phase of the cell cycle and the ^35^S-labelling of CAT (Fig. 5AB) indicating a cell cycle dependent accumulation of *de novo *synthesized CAT in promastigotes transfected with pXCAT3'H2A. Quantification of the radioactive signal revealed a 2-fold increase in the S phase (Fig 5B). Interestingly, the ratio between the *de novo *synthesis and number of cells in the S phase is similar to that obtained for the pXCAT5'III/3'I transfectants (0.66 ± 0.12). These results are in agreement with the *CAT *mRNAs polysome profiles showed in Fig. 5C. Most *CAT *mRNAs were not bound to functional ribosomes in the G1 phase (12% of the *CAT *transcripts), but shifted to polyribosome fractions during the S phase (48% of the *CAT *mRNAs). Remarkably, the position of the secondary peak in the central fractions in S phase blot (located in fractions 5–8), was similar to that obtained for the H4 transcripts and to that described for the endogenous H2A transcripts [11].

Thus, it can be concluded that a sequence segment located in the 3' UTR region of the *H2A4 *gene contains the *cis*-sequence element(s) responsible for the cell cycle regulated translation reported for the *Leishmania *histone genes.

### Analysis of the implication of the 5' UTR in the cell cycle regulated translation

To elucidate the role of the histone *H2A *5' UTR in the S phase dependent translation of the *CAT *gene we made a new construct in which the *CAT *reporter gene was placed between the 5' regulatory regions of *H2A4 *gene and the 3' regulatory regions of *HSP83 *gene. The construct, named pXCAT5'H2A (Fig. 1C), gave rise to a single *CAT *transcript of approximately 1.7 Kb in a Northern blot performed with total RNA from stably transfected cells lines containing this construct (Fig. 1D). Remarkably, the steady state level of CAT transcripts obtained from this cell line were lower to that observed in cell lines transfected with plasmids bearing both H2A regulatory regions (pXCAT5'III/3'I) or the 3'UTR alone (pXCAT3'H2A) (Fig. 1D).

**Figure 6:**
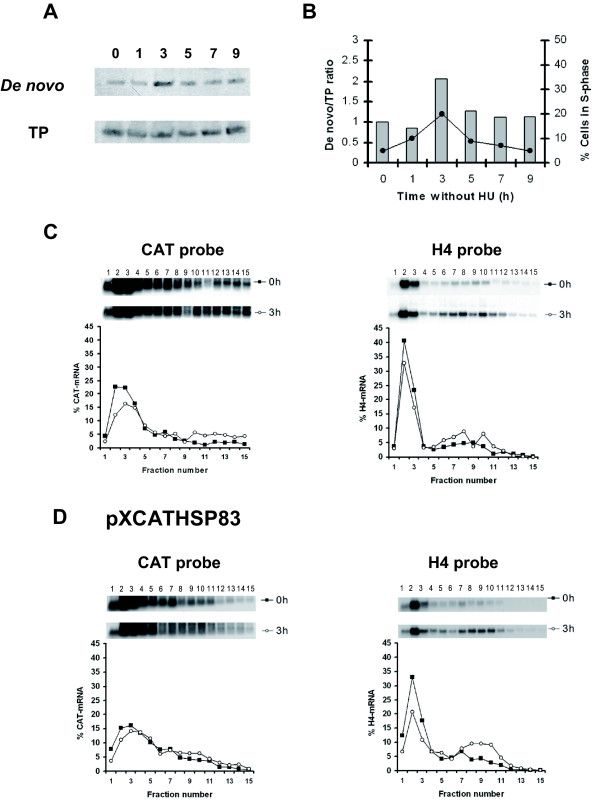
**Effect of the *H2A4 *5' UTR on regulation of CAT expression**. (A) Protein extracts from ^35^S-labelled *L. infantum *promastigotes transfected with pXCAT5'H2A and treated with 5 mM HU either for 12 h (lane 0) or at the indicated time period (in h) after removal of the drug, were used to immunoprecipitate CAT. After immunoprecipitation, protein samples were separated by SDS/PAGE and transferred on to a nitrocellulose membrane. Autoradiography (*De novo *panel) or Western blot with anti-CAT antibodies to reveal the total amount of CAT present in the samples (TP panel) is shown. (B) Densitometric analysis showing the ratio between the *de novo *synthesis of CAT and total amount of CAT (columns). The percentage of cells in the S phase is also included (black line). Northern blots prepared with RNA samples separated by sucrose gradients from promastigotes transfected with pXCAT5'H2A (C) or pXCATHSP83 (D) treated 12 with HU (lane 0) or 3 h after drug removal were sequentially hybridized with the CAT and the H4 probe. The autoradiographic exposure of the blots and the densitometric analysis is shown. Data correspond to one representative experiment of three independent assays with similar results.

Immunoprecipitation experiments with promastigotes transfected with the pXCAT5'H2A plasmid showed an increase in the metabolic labelling of CAT at 3 h after HU release (Fig. 6A). Densitometric analysis showed a 2-fold increase in the labelling of CAT when cells enter into the S phase (Fig. 6B). However, analysis of the polysomal profiles of cells transfected with pXCAT5'H2A revealed that *CAT *transcripts were actively translated at the G1 phase since they were associated with polyribosomal fractions (27% of the *CAT *mRNAs) at time 0 h after HU release (Fig. 6C). It was also observed that a higher percentage of transcripts were located in the polyribosomal fractions (44% of the *CAT *mRNAs) when cells progress into the S phase. In addition, the location of the reporter mRNAs in the bottom fractions of the sucrose gradient indicates a high translation rate for transcripts bearing the combination of the H2A 5' UTR and the *CAT *coding region in both cell cycle G1 and S phase. Subsequently, we analyzed the polysome profiles of the *CAT *transcripts in parasites transfected with the pXCATHSP83, a cell line that did not show cell cycle regulated expression of CAT. The results indicate that similar percentages of these transcripts were located into polysomes in the G1 (45% of the CAT mRNAs) and in the S phase (50%) (Fig. 6D). Contrary to *CAT *transcripts from the pXCAT5'H2A cell line, *CAT *transcripts from parasite transfected with PXCATHSP83 were in a similar active translational status along the cell cycle. Thus, it can be concluded that when the 5' UTR region of the *H2A4 *gene is located upstream of the reporter gene the translation level of *CAT *transcripts is increased in the S phase.

**Figure 7:**
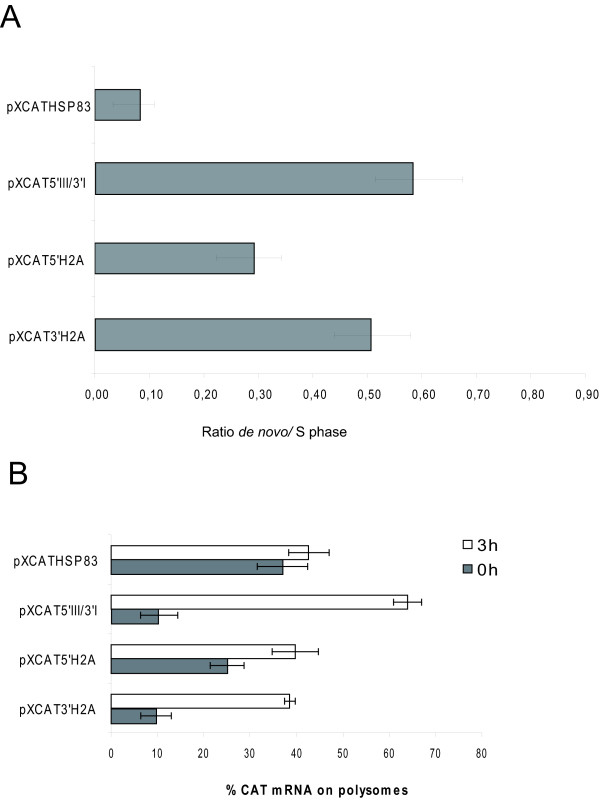
**Analysis of the CAT cell cycle regulated expression in different constructs**. (A) Ratio between the *de novo *CAT synthesis (normalised to the total amount of CAT) and number of cells in the S phase (3 h after HU removal) determined by three independent assays in cell lines transfected with pXCATHSP83, pXCAT5'III/3'I, pXCAT5'H2A and pXCAT3'H2A constructs. (B) Graphic showing the percentages of *CAT *mRNA on polysomes in the same cell lines. Data were obtained from the densitometric analysis of the blots performed with the RNAs separated on sucrose gradients from cell lines transfected with pXCATHSP83, pXCAT5'III/3'I, pXCAT5'H2A and pXCAT3'H2A constructs at G1 and S phase for three independent assays.

Taking into account the variations observed in the percentages of cells in the S-phase between experiments, three independent assays were performed with the cell lines transfected with pXCATHSP83, pXCAT5'III/3'I, pXCAT5'H2A and pXCAT3'H2A constructs. The ratio between the *de novo *synthesis of CAT (normalised to the total amount) and the amount of cells in the S phase in the different transfected cell lines was determined (Fig. 7A). An increase was observed in this ratio in both the pXCAT5'H2A and pXCAT3'H2A cell lines compared with pXCATHSP83 cell line. In addition, the ratio obtained with the parasites transfected with pXCAT3'H2A was higher than that observed for parasites transfected with pXCAT5'H2A. This ratio closely resembles the *de novo *synthesis ratio observed in the parasites transfected with the construct containing the two H2A regulatory regions (pXCAT5'III/3'I). In the same cell lines the percentage of *CAT *mRNA on polysomes was also analyzed (Fig. 7B). The distribution of the *CAT *transcripts on polysomes in parasites transfected with pXCAT3'H2A and pXCAT5'III/3'I constructs showed similar profiles, whereas a higher percentage of *CAT *transcripts was found on polysomes in the G1 phase in the pXCAT5'H2A transfected cell line. Altogether, these data indicate that although the 5' UTR and 3' UTR regions of the *H2A *gene are implicated in protein synthesis regulation, the 3' UTR plays the major role in the observed cell cycle dependent translation.

**Figure 8:**
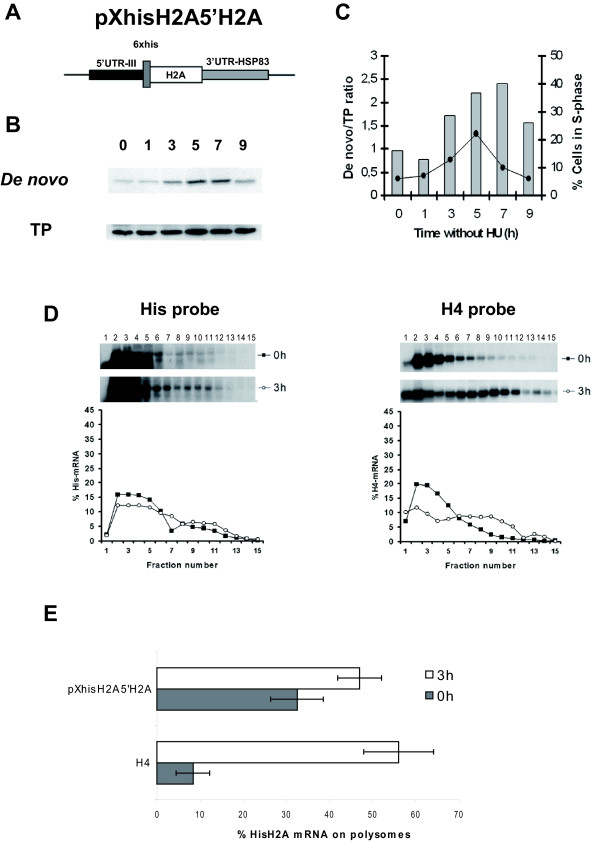
**Preferential translation induced in the S phase by the H2A4 5'UTR is maintained when a Histidine tagged H2A was employed as a reporter**. (A) Schematic representation of the pXhisH2A5'H2A construct. The position of the 6 × Histidine tag coding region is indicated. (B) Immunoprecipitation of the His-H2A of nuclear protein extracts from ^35^S-labelled *L. infantum *promastigotes transfected with pXhisH2A5'UTRH2A and treated with 5 mM HU either for 12 h (lane 0) or at the indicated time period (in h) after removal of the drug. The autoradiography (*De novo *panel) and western blot performed with the anti-His monoclonal antibody (TP panel) is shown. Data correspond to one representative experiment of two independent assays with similar results. (C) Densitometric analysis showing the ratio between de novo synthesis of His-H2A and total amount of His-H2A (columns). The percentage of cells in the S phase is also included (black line). (D) Northern blots prepared with RNA samples separated by sucrose gradients from promastigotes transfected with pXhisH2A5'H2A and treated 12 h with HU (lane 0) or 3 h after drug removal were sequentially hybridized with the 6 × His and the H4 probe. The autoradiographic exposure of the blots and the densitometric analysis is shown. Data correspond to one representative experiment of two independent assays with similar results. (E) Graphic showing the percentages of *His-H2A *and H4 mRNAs on polysomes at G1 and at the S phase obtained from two independent assays.

Whereas the expression of CAT is regulated in a similar form to that observed for the endogenous H4 histone in the cell line transfected with pXCAT3'H2A construct, in the pXCAT5'H2A cell line *CAT *transcripts are translated in the G1 phase although an increased translation was observed in the S phase of the cell cycle. Taking into account that the coding region of different reporter genes may affect the translational efficiency in kinetoplastids [30] we decided to study the regulatory role of the *H2A *5' UTR with an His-tagged H2A as reporter gene, instead of CAT. Therefore, we created a new construct named pXhisH2A5'H2A in which the sequence coding for six Histidine residues, followed by the *H2A4 *coding region was flanked by the 5' regions derived from gene *H2A4 *and 3' regions derived from *HSP83 *gene (Fig 8A). The *de novo *synthesis of His-H2A was determined by immunoprecipitation using an anti-*His *monoclonal antibody. This was performed on nuclear protein extracts obtained from cells progressing along the cell cycle and labelled with [^35^S]methionine. In Fig. 8B–C, the autoradiograph exposure of the blot and the densitometric analysis obtained in one of the two independent experiments are shown. An accumulation of the *de novo *synthesized His-H2A at the S phase was observed. The ratio between the *de novo *synthesis of the His-H2A protein and number of cells in the S phase (0.35 ± 0.12) is similar to that obtained for CAT protein in cell line transfected with pXCAT5'H2A (0.29 ± 0.09). However, it was also shown that the *de novo *synthesis of His-H2A was maintained when cells progressed into the G2/M phase. The polysomal profiles of the *His-H2A *transcripts have been determined in cells transfected with the pXhisH2A5'H2A construct in the G1 and the S phase (Fig. 8D). The results of two independent assays revealed an active translation of these transcripts during the G1 phase (32.4 ± 6.09) with an increase in the percentage of transcripts located in the secondary peak 3 h after HU release (47.1 ± 5.12) (Fig. 8E). On the other hand, and as a consequence of the replacement of CAT to His-H2A we observed that the position of the *His-H2A *mRNAs secondary peak on the sucrose gradient was similar to that observed for the *H4 *transcripts used as control of the endogenous expression (Fig. 8D).

These results reinforce the implication of the H2A 5' UTR, in addition to that of the 3' UTR, in the translation of the histone transcripts during the S phase of the cell cycle since for both reporters a slight increase was observed in the *de novo *synthesis and in the percentage of mRNAs located into the polysome fractions. The role of this region in the cell cycle expression of histone genes is related with an enhancement of translation initiation of H2A transcripts when these have access to the translational machinery.

## Conclusion

Changes in the steady-state levels of *Leishmania *regulated transcripts in response to different stimuli have been associated with post-transcriptional events controlling mRNA stability. This kind of regulation involves sequences present mainly in the divergent 3' UTRs, although sequences located at the 5' UTRs and in some cases on intergenic regions (IR) have also been implicated [23,31]. The presence of divergent sequences in the 3' UTR of genes coding for nearly identical proteins has been correlated with a differential pattern of expression of these genes during the parasite life cycle stages as this occurs for example with the promastigote major surface protease GP63 [32] or during heat shock as occurs in the *L. infantum HSP70 *gene family [33,34]. *L. infantum *histone *H2A *coding genes are organized in tandem as two independent clusters, and like many housekeeping genes they possess a high sequence conservation in the coding regions and high divergence in the 5' and 3' UTRs [25]. The data presented in this paper indicate that contrary to what has been shown for other gene families, the sequence differences in the 5' and 3' UTRs of *H2A *gene copies are not implicated in a differential expression of this gene. In fact, a similar S phase dependent, *de novo *synthesized CAT protein was detected in parasite cell lines stably transfected with different constructs containing distinct combinations of the histone *H2A *5' and 3' UTR regions.

In the last few years an increasing number of reports show that sequences located at the 3' UTR of different *Leishmania *genes are implicated in the regulation of mRNA translation (reviewed in [35]). It has been shown that a 450-nt long region within the amastin 3' UTR confers amastigote-specific gene expression by a mechanism that leads to an increase in mRNA translation [36]. This sequence is also present in the 3' UTRs of many protein coding genes that are specifically expressed in the amastigote stage of the parasite. During heat shock, the *HSP83 *3' UTR has been involved in the preferential translation of the *HSP83 *genes in *L. amazonensis *and *L. infantum *[28,37]. The data presented in this work show that in parasites transfected with the pXCAT5'III/3'I construct (containing the regulatory regions of gene *H2A4*) CAT expression is regulated mainly at the translational level in a cell cycle dependent manner similarly to the endogenous *Leishmania *histones [11]. In fact, in the transfected parasites there were not significant variations in the steady-state levels of *CAT *mRNAs along the cell cycle whereas drastic changes in the polysome profiles of histone mRNAs were observed during the progression from the G1 to the S phase, concomitant with an increase in the *de novo *synthesis of CAT in the S phase of the cell cycle. Furthermore, our data show that the *H2A *3' UTR is implicated in the cell cycle dependent translational control of CAT. The presence of the *H2A *3'UTR in the *CAT *transcripts was associated with the accumulation of the mRNAs in a translational incompetent state in the G1 phase. A similar accumulation of untranslated transcripts has been described for some *Leishmania HSP70 *bearing specific 3'UTR regions [33]. Most likely, these parasites accumulate untranslated transcripts in order to mount rapid responses to environmental changes. The characterization of the regulatory protein(s) that interact with the *H2A 3' UTR *requires the definition of the mRNA signals involved in the interactions. In *Leishmania *only a few post-transcriptional regulatory *cis*-regulatory elements have been described (recently reviewed in [31]), and many of them involve large regions on the 3' UTRs of the mRNAs. Interestingly, the existence of a defined conserved *cis *element in the 3' UTR of almost all *Leishmania *histone genes (CATAGA followed by a T box) has been described [38]. The presence of the CATAGA box in the 5' and 3' UTRs of some genes implicated with *Leishmania *DNA metabolism has been correlated with the accumulation of their mRNAs in the S phase of the cell cycle [39]. Whether or not this element plays a role in the translational control of the expression of the *Leishmania *histone proteins along the cell cycle requires further elucidation. Definition of the critical regulatory sequences located in the 3' UTR of the H2A genes will allow the characterization of the control mechanism operating in these transcripts (a translational repression out of the S phase or an induced translation in the S phase).

Little is known about the implication of 5' UTR regions in mRNA translation in *Leishmania*. The *Leishmania HSP83 *5' UTR was described to play a role in the preferential translation of parasite *HSP83 *during heat shock, that depends on the presence of the *HSP83 *3'UTR in the same transcripts [37]. Our data show that the *H2A *5' UTR has a minor contribution in the cell cycle dependent expression of histone H2A. Both, *CAT *and *His-H2A *transcripts bearing histone *H2A *5' UTR were translated at the G1 phase although they were more efficiently translated in the S phase. It has been reported that the translational efficiency in kinetoplastids can be affected by the reporter employed [30,40]. In our experiments, by changing the reporters differences were only observed when cells reached the G2/M phase. This indicates that further analysis should be done in order to clarify the role of this region in the cell cycle regulation of *Leishmania *histone genes. In higher eukaryotes sequence elements located in the 5' UTRs are usually involved in the interactions of factor(s) that down-regulate the level of translation (reviewed in [41,42]). On the other hand, 5' UTRs *cis *elements that allow the interaction of translational enhancers have also been described [43,44]. The mechanism underlying the 5' UTR mediated translation regulation of histone H2A 5' UTR is still unclear. Both, translational activators interacting in the S phase or translational repressors interacting out of the S phase could be responsible for the observed effects.

## Methods

### Parasites and cell cycle analysis

Promastigotes of *Leishmania infantum *(M/CAN/ES/96/BCN150) were grown at 26°C in RPMI 1640 medium (Gibco, Paisley, U.K.), supplemented with 10% (v/v) heat-inactivated foetal calf serum (ICN Pharmaceuticals, Basingstoke, Hants, U.K.). Experimental cultures were initiated at 1 × 10^6 ^promastigotes/ml and harvested for study in the exponential phase of growth (6 × 10^6 ^promastigotes/ml).

For the synchronization of DNA replication, parasites in the exponential phase of growth were treated with 5 mM hydroxyurea (HU) for 12 h. Then, parasites were harvested, washed twice with PBS and resuspended in fresh medium without the drug. Different phases of the *Leishmania *cell cycle were determined by flow-cytometric analysis of DNA content in promastigotes stained with propidium iodide as described [11].

### Polysome fractionation by sucrose gradients, RNA purification and Northern blot analysis

The analysis of the polysomal distribution of *CAT *mRNAs was performed by cytosolic fractionation in linear 15–40% (w/v) sucrose gradients as previously described [11]. After fractionation, RNA was separated on denaturing 1% agarose-formaldehyde gels and electrotransferred to nylon membranes using an LKB system (Amersham Biosciences).

Total *L. infantum *RNA was isolated from the different cell lines as described previously [45]. Northern blots were hybridized with a oligonucleotide complementary to the 3' terminal region of the coding region from the *CAT *gene (5'-TTACGCCCCGCCCTGCCACT-3') or a oligonucleotide complementary to the sequence coding for the 6 × His tag (5'-GTGATGGTGATGGTGATG 3') labelled at the 5' end using T4 polynucleotide kinase and gamma- [^32^P]ATP as described [46]. Hybridization was performed at 55°C in 6 × NET buffer (0.9 M NaCl, 6 mM EDTA, 0.5% SDS, 0.09 M Tris-HCl (pH 7.5), and 0.25 mg ml^-1 ^of herring sperm DNA). Five post-hybridization washes were performed in 6 × SSC buffer (0.9 M NaCl, 0.09 M sodium citrate) at room temperature. A final wash of 10 min was performed at 55°C in the same buffer. When indicated, and for re-use, blots were treated with 0.1% SDS for 15 min at 95°C to remove the previous probe and hybridized with a *L. infantum *histone H4 probe (clone LiH4-1 [19]) or a *L. infantum *histone H2A probe (clone rLiH2A-Ct-Q [47]) coding for the C-terminal 67 aminoacids of the H2A protein labelled by nick translation as described [48]. Hybridization was performed in 50% (v/v) formamide, 6 × SSC buffer (0.9 M NaCl, 0.09 M sodium citrate), 0.1% SDS and 0.25 mg ml^-1 ^of herring sperm DNA at 42°C overnight. Final post-hybridization washes were performed in 0.1 × SSC (15 mM NaCl, 1.5 mM sodium citrate) plus 0.2% SDS at 50°C for 1 h.

### Plasmid constructs and parasite transfection

Different constructs were generated using regulatory regions derived from the *L. infantum H2A *gene locus 2 containing *H2A4, H2A5 and H2A6 *genes (accession number AJ419627). Clone pBls5'UTR-II containing a DNA fragment that includes the 5'UTR from gene *H2A5 *along with upstream sequences (including the 3' UTR from gene *H2A4 *and the intergenic region) was obtained by PCR using the following primers: forward, 5'-TCCCCCGGGGTCCTCCGGCCTGACAGCGC-3'; reverse, 5'- GCGATATCCATGGTTGCGGAAAGGAGAG-3' (underlined are restriction sites included in the primers to facilitate cloning). The PCR product was digested with *Cfr*9I and *Eco*RV restriction enzymes (cut sites underlined) and cloned in pBluescript KS(-) (pBls). An *Eco*RI and *Eco*RV DNA clone, named pBls5'UTR-III, containing the 5'UTR from gene *H2A4 *along with upstream sequence (600 nt upstream the putative AG *trans-*splicing acceptor site) was also obtained by PCR using the following primers: forward, 5'-GGAATTCAACAATGCAGGGAA-3'; reverse, 5'- GCGATATCCATGGCTGCGATGGGTAGGT-3'. The 3' UTR and downstream sequences from gene *H2A5 *(including the intergenic region and the 5' UTR from gene *H2A6*) were PCR amplified using the following primers: forward, 5'-CCCAAGCTTAGCACACCTACCCCTCTCTT-3'; reverse 5'-GCGTCGACGGATCCCATGGCTGCGATGGGTAGGT). The PCR insert was digested with *Hind*III + *Sal*I and cloned in pBls (clone pBl3'UTR-III). The 3' UTR and downstream sequences derived from gene *H2A4 *(including the intergenic region and the 5' UTR from gene *H2A5*) was PCR amplified using the following primers: forward, 5'- CCATCGATGTCCTCCGGCCTGACAGCGC-3'; reverse, 5' GCGTCGACGGATCCCATGGTTGCGGAAAGGAGAC-3'. The PCR insert was digested with *Cla*I and *Sal*I and cloned in pBls (clone pBl3'UTR-I).

As control, the region 3'UTR+IR+5'UTR located between coding regions of genes 1 and 2 of the *L. infantum HSP83 *gene cluster was employed [27]. For that purpose this region was PCR amplified including different restriction enzyme cut sites. When this region was placed upstream the reporter gene the following primers were employed: forward, 5'- CGGGATCCCGCGCACTGCTCTTTACAT-3'; and reverse 5'-TGCCTGCAGCGTCTCCGTCATGGTTGCAG-3'. The PCR product was digested with *Bam*HI and *Pst*I and cloned in pBls (clone pBls5'UTR-HSP83). When this region was placed downstream the reporter gene the following primers were used: forward, 5'-CGAAGCTTAGGTGGACTGAGCCGGTA-3'; and reverse 5'-GCGTCGACGGATCCGCTCTCCGTCATGGTTGCAG-3'. The PCR product was digested with *Hind*III and *Sal*I and cloned in pBls (clone pBls3'UTR-HSP83). Both clones were generously provided by Dr Ruth Larreta.

Next we made constructs in which the inserts of the different plasmid described above were combined controlling the expression of the *CAT *coding region. All plasmid constructs were first generated in a pBluescript KS (-) containing the *CAT *coding region (pBlCAT clone [28]) and cloned after digestion with *Bam*HI into the *Leishmania *expression plasmid pX63NEO [26]. DNA inserts containing the *CAT *gene and the 5'- and 3'-regulatory regions were cloned in the opposite orientation with respect the *NEO *gene contained in the pX63NEO plasmid (Fig. 1B) to generate the following constructs (Fig. 1C):

pXCAT5'III/3'I in which *CAT *gene is flanked by both regulatory regions of gene *H2A4*.

pXCAT5'III/3'III in which *CAT *gene is flanked by the 5'-regulatory regions of gene *H2A4 *and the 3'-regulatory regions of gene *H2A5*.

pXCAT5'II/3'I in which *CAT *gene is flanked by the 5'-regulatory regions of gene *H2A5 *and the 3'-regulatory regions of gene *H2A4*.

pXCAT5'II/3'III in which *CAT *gene is flanked by the 5'- and 3'-regulatory regions of gene *H2A5*.

pXCAT3'H2A in which *CAT *gene is flanked by the 5'-regulatory regions of the *HSP83 *and the 3'-regulatory regions of gene *H2A4*.

pXCAT5'H2A in which *CAT *gene is flanked by the 5'-regulatory regions of gene *H2A4 *and the 3'-regulatory regions of gene *HSP83*.

pXCATHSP83 in which *CAT *gene is flanked by the regulatory regions of gene *HSP83*.

For the analysis of the implication of the *H2A4 *5' UTR in the preferential translation during the cell cycle a DNA coding for a 6 × Histidine tagged H2A was obtained by PCR using the following primers: forward 5'-GCGATATCATG*CATCACCATCACCATCAC*GCTACTCCTCGCAGCGCCAA-3' (the sequence coding for the 6 × His tag is indicated in italics) and reverse 5'-CCCAAGCTTACGCGCTCGGTGTCGCCC-3'. The PCR insert was digested with *EcoR*V and *Hind*III and cloned in pBls. The inserts of clone pBls5'UTR-III and clone pBls3'UTR-HSP83 were subcloned upstream and downstream the *His-H2A *insert, respectively. After digestion with *Bam*HI, the DNA insert containing the *His-H2A *coding region and the 5'- and 3'-regulatory regions were cloned in the opposite orientation with respect the *NEO *gene contained in the pX63NEO plasmid generating the pXhisH2A5'H2A clone

For the transfections, plasmids DNAs were obtained with the Plasmid Maxy Kit (Qiagen, Hilden, Germany). Parasites in the exponential phase of growth were harvested by centrifugation and resuspended at a density of 10^8 ^parasite/ml in ice-cold EPB buffer (21 mM Hepes (pH 7.5), 137 mM NaCl, 5 mM KCl, 0.7 mM Na_2_HPO_4_, and 6 mM glucose). Afterwards, 0.4 ml of cell suspension were put into a 0.4-cm electroporation cuvette and chilled on ice for 10 min. Plasmid DNA (10 μg) were added and the cells were pulsed two times (500 μF, 2.25 kV/cm) using a Bio-Rad Gene Pulser with a capacitance extender module. Afterwards, samples were transferred to 10 ml of RPMI medium supplemented with 20% FCS and incubated for 24 h at 26°C. After incubation in medium lacking antibiotics, transfected cell lines were selected in the presence of 20 μg/ml of geneticin (G418; Roche Diagnostics, Mannheim, Germany). Stable cultures were obtained after 25–30 days of incubation at 26°C. Stably transfected cell lines containing the *CAT *reporter gene were examined for the presence of *CAT *transcripts by Northern blot (Fig. 1D)

### Mapping of RNA ends of the CAT transcripts by RT-PCR

To determine the sites of poly(A) and splice leader addition in the *CAT *transcripts in the different constructs, cDNAs were generated by reverse transcription of total RNA extracted from the different cell lines. A (dT)_18 _primer that included the *Cla*I restriction sequence (5'-CCATCGATTTTTTTTTTTTTTTTTT-3') namely CdT, was employed for reverse transcription of the transcripts containing the 3' UTR of gene *H2A4*. A (dT)_18 _primer that included the *Hind*III restriction sequence (5'-CCAAGCTTTTTTTTTTTTTTTTTT-3'), namely HdT was employed for reverse transcription of the transcripts containing the 3' UTR of gene *H2A5 *and gene *HSP83*. Reverse transcriptions steps were performed with SuperScript™ II reverse transcriptase (Invitrogen, Carlsbad, CA, U.S.A.) following the manufacturer's protocol (2 μg of total RNA per reaction). To map the 5' UTR end of the *CAT *transcripts an aliquot of the different cDNAs was PCR amplified using the following primers: forward, 5'-GCGATATCGCTATATAAGTATCAGTTTCTGTAC-3' (underlined the *EcoR*V cut site) derived from the *Leishmania *splice leader sequence; reverse, 5'- CCCAAGCTTACGCCCCGCCCTGCCACT -3' (underlined the *Hind*III cut site) derived from the *CAT *gene coding region. The obtained PCR inserts were directly sequenced using a primer reverse and complement to the nucleotides 308–327 of the *CAT *gene coding region (5'-GTATTCACTCCAGAGCGATG-3'). To map the 3' UTR end of the *CAT *transcripts a forward primer derived from the first nucleotides coding for the CAT protein (5'-GCGATATCATGGAGAAAAAAATCACTGG-3') was employed in combination with CdT or HdT. The PCR inserts containing the *H2A4 *or *H2A5 *3'UTRs were directly sequenced using a primer derived from nucleotides positions 309–328 of the *CAT *gene coding region (5'-ATCGCTCTGGAGTGAATACC-3'). For the sequencing of the 3' boundaries of *CAT *transcripts containing the 3' UTR of gene *HSP83*, a primer derived from nucleotides 661–670 of this sequence (5'- CGGCGTGGCAGATCGGGATG- 3') was employed.

In all constructs containing the 5' UTR from gene *H2A4 *the trans-splicing acceptor site was located 66 nucleotides upstream from the beginning of the *H2A4 *coding region (position 1611 of the H2A Locus 2 [25]; GenBank™/EBI Data Bank accession number AJ419627). In all constructs containing the 5' UTR from gene *H2A5 *the trans-splicing acceptor site was located 105 nucleotides upstream from the beginning of the *H2A5 *coding region (position 2609 of the H2A Locus 2 [25]; GenBank™/EBI Data Bank accession number AJ419627). In the two constructs containing the 5'UTR from gene *HSP83 *the trans-splicing acceptor site was located 290 nucleotides upstream from the beginning of the *HSP83 *coding region (GenBank™/EBI Data Bank X87770[28]) the same position described for the endogenous *HSP83 *transcripts [28]. In all constructs containing the 3' UTR from gene *H2A4 *the polyadenilation site of the 3' UTR was determined to be at nucleotide 211 from the stop codon (position 2286 of the H2A Locus 2 [25]; GenBank™/EBI Data Bank accession number AJ419627). In all constructs containing the 3' UTR from gene *H2A5 *the polyadenilation site of the 3' UTR was determined to be at nucleotide 305 from the stop codon (position 3418 of the H2A Locus 2 [25]; GenBank™/EBI Data Bank accession number AJ419627). In the two constructs containing the 3' UTR from gene *HSP83 *the polyadenilation site of the 3' UTR was determined to be at nucleotide 867 from the stop codon (GenBank™/EBI Data Bank X87770[28]), 29 nucleotides upstream to the position reported for the endogenous *HSP83 *transcripts. Thus, although an alternative sequence was employed in the *CAT *transcripts bearing the HSP83 3'UTR, the length of this region is identical in both pXCATHSP83 and pXCAT5'H2A constructs.

### Metabolic labelling of proteins, immunoprecipitation and western blotting

For protein labelling, 10 ml aliquots of promastigotes at the exponential phase of growth were removed at different time periods after HU removal, washed twice with PBS and resuspended in 100 μl of Dulbecco's medium (without Methionine and Cysteine) supplemented with 7 μl of the Pro-mixTM ^35^S *in vitro *cell-labelling mix containing L- [^35^S]methionine and L- [^35^S]cysteine (1 mCi ml^-1 ^and 1000 Ci mmol^-1 ^respectively; Amersham Biosciences), during 60 min at 26°C. The labelling medium was also supplemented with 5 mM HU for aliquots taken at zero time. Analysis of the *de novo *CAT synthesis was performed on ^35^S-labelled promastigotes (5 × 10^7 ^cells) by immunoprecipitation with an anti-CAT rabbit polyclonal serum (Sigma-Aldrich, St. Louis.MO) as described in [28]. Analysis of the novo His-H2A synthesis was performed on ^35^S-labelled promastigotes (5 × 10^7 ^cells) by immunoprecipitation with an anti-His mouse monoclonal antibody (Sigma-Aldrich, St. Louis. MO). For that purpose, parasite nuclear fractions were extracted after labelling as described [49]. Briefly, labelled promastigotes were pelleted, washed twice in ice-cold PBS, resuspended in 400 μl of prechilled buffer A (10 mM Hepes (pH 7.5), 10 mM KCl, 0.1 mM EDTA, 0.1 mM EGTA, 1 mM Dithiothreitol, 0.5 mM PMSF), and incubated for 15 min on ice. After incubation, Nonidet P40 was added to a final concentration of 0.6%, and cells were lysed by vigorous vortex-mixing for 10 s, and immediately pelleted in a microfuge (13000 g). The pelleted nuclei were resuspended in 100 μl of lysis buffer (1% SDS, 10 mM EDTA (pH 8.0), 50 mM Tris HCl (pH 8.0), 150 mM NaCl, 1% Triton X-100, 1 mM PMSF, 8 μg ml^-1 ^Leupeptin, 4 μg ml^-1 ^Pepstatin, 4 μg ml^-1 ^Aprotinin) and sonicated for 30 min in a water bath at 4°C for clearance of nuclei acids. After lysis, samples were microfuged for 15 min and the supernatant was diluted in 675 μl of ChIP buffer (0.01% SDS, 1.1% Triton X-100, 1.2 mM EDTA (pH 8.0), 16.7 mM Tris-HCl (pH 8.1), 167 mM NaCl,1 mM PMSF, 8 μg ml^-1^, Leupeptin, 4 μg ml^-1 ^Pepstatin, 4 μg ml^-1 ^Aprotinin), mixed with 3 μl of anti-His monoclonal antibody (Qiagen, Hilden, Germany) and incubated on an orbital rotator for 15 h at 4°C. Agarose beads (15 μl) conjugated with Protein G (Sigma) were equilibrated in 50 μl of ChIP buffer added to the *Leishmania *nuclear extract/anti His monoclonal antibody mixture. The mixture was incubated on a rotator for 2 h at 4°C. The beads were collected by centrifugation and washed three times with 0.5 ml of buffer A (10 mM Tris-HCl (pH 8.0), 30 mM NaCl, 2% Triton X-100), twice in 0.5 ml of buffer B (10 mM Tris-HCl (pH 8.0), 50 mM NaCl, 2% Triton X-100) and once in 0.5 ml of buffer C (10 mM Tris-HCl (pH 8.0) 0.05% Triton X-100). Finally, the beads were resuspended in 60 μl of 2 × Laemmli's buffer.

Immunoprecipitated proteins were resolved by SDS-PAGE on 10% acrylamide gels and transferred on to nitrocellulose membranes (Amersham Bioscience, Aylesbury, UK) in a Mini-protein system (Bio-Rad Laboratories, Hercules, CA, U.S.A.) and exposed to an autoradiographic film. Total amount of immunoprecipitated CAT and His-H2A was analysed by Western blotting using the anti-CAT serum or the anti-His monoclonal antibody. As secondary antibody an anti-rabbit IgG-peroxidase immunoconjugate or anti-mouse IgG-peroxidase immunoconjugate (Nordic Immunologic, Tilburg, The Netherlands) was used. The specific binding was revealed with the ECL^® ^Western-blotting detection reagent (Amersham Biosciences).

### Quantitative analysis

The autoradiographs were scanned with a laser densitometer (Image QuantTM version 3.0; Molecular Dynamics). Measurements were performed under conditions in which a linear correlation existed between the amount of proteins or RNA and the band intensity on the autoradiographs.

## Abbreviations

CAT: Chloramphenicol Acetyltransferase; DHFR-TS: Dihydropholate Reductase-Thymidylate Synthase; HSP: heat shock protein; UTR: untranslated regions; HU: Hydroxyurea; UPR: upstream regions; DNR: downstream regions; pBls: pBluescript KS(-).

## Authors' contributions

DRA and MS carried out the construction of the transfection plasmids and performed the immunoprecipitation assays shown in Fig. 2, 5, 6 and 7. LR performed the mapping of RNA ends by RT-PCR. DRA performed the Northern blots (Fig. 1 and 4) and the analysis of RNA fractionation in sucrose gradient shown in Fig., 4, 5, 6, 7 and 8. SI performed the determination by flow-cytometric analysis of DNA content in promastigotes stained with propidium iodide shown in Fig. 2, 4, 5, 6, 7 and 8. KS critically read the manuscript. PB participated in the experimental design and read and edited the manuscript. VMG and CA gave laboratory support and critically read the manuscript. All authors read and approved the final manuscript.
